# Automated cleaning of fan coil units with a natural detergent-disinfectant product

**DOI:** 10.1186/1476-0711-9-29

**Published:** 2010-10-12

**Authors:** Giorgio Liguori, Maria Bagattini, Francesca Gallè, Mario Negrone, Valeria Di Onofrio, Maria Triassi

**Affiliations:** 1 Cattedra di Igiene ed Epidemiologia, Università degli studi di Napoli "Parthenope", Napoli, Italy; 2 Dipartimento di Scienze Mediche Preventive, Sezione di Igiene, Università degli studi di Napoli "Federico II", Napoli, Italy; 3 Servizio di Igiene degli alimenti e della nutrizione, Dipartimento di Medicina Preventiva, Agenzia di Sanità Pubblica, Potenza, Italy

## Abstract

**Background:**

Air conditioning systems represent one important source of microbial pollutants for indoor air. In the past few years, numerous strategies have been conceived to reduce the contamination of air conditioners, mainly in hospital settings. The biocidal detergent BATT2 represents a natural product obtained through extraction from brown seaweeds, that has been tested previously on multidrug-resistant microorganisms.

**Methods:**

BATT2 has been utilized for the disinfection of fan coil units from four air conditioning systems located in hospital environments with a mean degree of risk. Samples were collected from the air supplied by the conditioning systems and from the surfaces of fan coil units, before and after sanitization procedures. Total microbial counts at 37°C and 22°C and mycotic count at 32°C were evaluated. *Staphylococci* and *Pseudomonas aeruginosa *were also detected on surfaces samples.

**Results:**

The biodetergent was able to reduce up 50% of the microbial pollution of fan coil units surfaces and air supplied by the air conditioners.

**Conclusions:**

BATT2 could be considered for cleaning/disinfection of air conditioning systems, that should be performed on the basis of accurate and verifiable sanitization protocols.

## Introduction

Microbial contamination of air has become of interest in the past two decades because of the correlation of sick building syndrome (SBS) with indoor air pollution [1,2]. In fact, the onset of SBS, which comprises a series of symptoms such as eye irritation, airways dryness, headache, sleepiness, and skin rash and itch, seems to be related to the presence of microbes or their components in indoor air [3-7]. Bio-contamination has the same harmful effects as chemical pollutants on the health of individuals [3]. This is important in hospital settings, especially for those units that accommodate patients with high-risk conditions [8].

The main sources of microbial pollutants for indoor environments are people and air conditioning systems, which allow the survival and multiplication of microorganisms [9,10]. The dampness inside these systems, together with incorrect management of sanitization procedures, can promote the spread of microorganisms and cause transmission of many infectious diseases [4,7,9,10].

In the past few years, the need to contain biological pollution in indoor environments has led to numerous strategies for the reduction of contamination of air conditioning systems, mainly in hospitals and in those settings where the absence of microorganisms is required [11-14].

The biocidal detergent NTI 60 C428 CLEANER COND. B (Natural Technologies Italia srl) (here reported as BATT2) is a natural product that is obtained through extraction from brown seaweed. Its cleaning/disinfectant effect is based on the synergistic action of natural vegetable extracts combined with an amphionic alkyl-amino carboxylate and alkyl betaine. The effectiveness of this product has been demonstrated by its effects on multidrug-resistant nosocomial microorganisms [15].

The aim of the present study was to analyze the possibility of employing this product for cleaning and disinfection of fan coil units from air conditioners placed in hospital laboratory rooms, whose activities are considered at mean degree of risk [8]. This was achieved by evaluating the ability of BATT2 to reduce bio-contamination of fan coil unit surfaces and the air delivered by the air conditioning systems.

## Materials and methods

Four air-cooled two-pipes fan coil units were chosen for the study. They were placed in four different rooms (A-D) of the Laboratory of Microbiology, Department of Medical Preventive Sciences, University of Naples "Federico II". These rooms are used as a reception, filtration room, laboratory for *Legionella *spp. detection, and virology laboratory, respectively. The air conditioners were chosen according to the local representative of Natural Technologies Italia srl, by evaluating the similarity of their technical and structural features and making sure that routine 6-monthly maintenance procedures, based on the use of an alcool-based detergent, were not underway. The fan coil units in the first three rooms (A-C) were considered for the tests, while that located in the virology laboratory (D) was chosen as a control because of its cleaner conditions, due to the lower number of persons who normally work in this room.

The periodical sanitization of air conditioners with BATT2 was carried out through automated systems for fan coil unit cleaning, which has been patented as "Unit H. E. C. & I." by Natural Technologies Italia srl. These devices allow the supply of a specified amount of atomized product at planned time intervals. As recommended by the manufacturer, each cleaning/disinfection intervention was performed with 30-40 ml detergent at a 25% concentration, supplied for 5 s, at 06:00 h on each sampling day.

The monitoring program was carried out in two phases: (1) microbiological samples were collected to evaluate the normal level of bio-contamination of the fan coil units, without any sanitization treatment; and (2) the sampling was performed after periodical automated sanitization with BATT2. Each phase extended for 4 weeks and included 10 non-consecutive sampling days. Samples were collected from the air supplied by the conditioning systems and from the surfaces of the fan coil units. For air samples, total microbial count (TMC) at 37°C and at 22°C and mycotic count at 32°C were determined. Detection of airborne microorganisms was carried out on the basis of UNI EN 13098:2002 recommendations [16]. Air samples were collected through the active sampler Surface Air System (SAS, PBI International), which conveyed the air on RODAC plates with: (1) Standard Plate Count (SPC) agar APHA (Oxoid Italia SpA) for microbial counts; and (2) Sabouraud Dextrose agar (Oxoid Italia SpA) for the detection of yeasts and moulds. The time of incubation was 24 h for mesophilic count and 72 h for psychrophilic count and mycotic count. For each sample, a volume of 1,080 l was collected. The sampler was held over each fan coil unit, at about 1.5 m from the floor [17,18]. Results were expressed as CFU/m^3^.

As for the surfaces, the level of bio-contamination was determined by detection of the following parameters [17]: (1) total microbial count at 37°C; (2) coagulase-negative *Staphylococci* and *Staphylococcus aureus*; (3) *Pseudomonas aeruginosa*; and (4) yeasts and moulds. Surface sampling was performed using RODAC plates, by employing the following culture media: (1) SPC agar APHA (Oxoid Italia SpA), for TMC; (2) Mannitol Salt Agar (Oxoid Italia SpA) for the detection of *Staphylococci*; (3) MacConkey agar No. 3 (Oxoid Italia SpA) for the detection of *P. aeruginosa*; and (4) Sabouraud Dextrose agar (Oxoid Italia SpA) for the mycotic count. Incubation conditions were 24 h at 37°C for mesophilic count, 24-48 h at 37°C for *Staphylococci* and *P. aeruginosa *and 72 h at 32°C for mycotic count. Both right and left sides of the fan coil units were sampled, with a total number of 20 samples for each parameter. Results were expressed as CFU/25 cm^2^. The identification of microorganisms was carried out through API galleries (bioMérieuxItalia SpA).

All the tests were performed on the morning before the start of the activities normally carried out in each room. Fan coil units were started up early, after about 1 h from the automated sanitization procedure, when this was carried out. Air and surfaces samples were collected after nearly 15 min from air conditioners switch on. To avoid outside contamination, doors and windows were kept closed until the sampling time [17].

For the air conditioner in room D, which was chosen as the reference system, only the first phase of monitoring, without any sanitization treatment, was performed. The same sampling procedure and times described above were used.

Statistical analysis was based on Student's *t *test and was carried out with SPSS for Windows version 12. A p value < 0.05 was considered significant.

## Results

The mean mesophilic microbial count of the air supplied by the three conditioning systems in rooms A-C decreased by 73.2%, 52.2% and 69.2%, respectively after treatment with BATT2 (Table 1). With regard to psychrophilic count, there was a reduction of 79.8% for room A, 65.8% for B and 62% for C. There were significant differences in the mesophilic and psychrophilic microbial counts of the air before and after the use of BATT2 (p < 0.05), with the only exception of the mesophilic count of room B (Table 1).

**Table 1:** Differences among mean microbial counts registered for air samples before (T_1_) and after (T_2_) sanitization with BATT2 (n. of samples: 10 for each mean value).

Room	**Mean value T**_**1 **_**± D.S. (CFU/m**^**3**^**)**	**Mean value T**_**2 **_**± D.S. (CFU/m**^**3**^**)**	Variation (%)	Student's t	p
**A - TMC 37°C**	25,7 ± 19,4	6,9 ± 8,4	-73.2	2,80	0,0116

**B - TMC 37°C**	15,7 ± 10,5	7,5 ± 7,5	-52.2	2,010	0,0596

**C - TMC 37°C**	13,3 ± 12,4	4,1 ± 3,7	-69.2	2,25	0,0369

**A - TMC 22°C**	124,3 ± 91,4	25,1 ± 13,9	-79.8	3,39	0,0032

**B - TMC 22°C**	78,7 ± 67,7	26,9 ± 27,6	-65.8	2,24	0,0379

**C - TMC 22°C**	46,3 ± 38,3	17,6 ± 12,5	-62	2,25	0,0369

As for the surfaces of rooms B and C, the mean percentage reduction in microbial count was 91.1% and 78.5%, respectively (Table 2). Similar decreases were observed for coagulase-negative *Staphylococci* isolated (96.8% for room B and 98.5% for C) (Table 3). With regard to the air conditioner surfaces of room A, the mean percentage decrease in microbial count was 25%, with an increase in *Staphylococci* (from 0.2 to 0.26 CFU/25 cm^2^) after sanitization with BATT2. However, these differences were not significant (Tables 2 and 3).

**Table 2:** Differences among mean microbial counts registered for fan coil units surfaces before (T_1_) and after (T_2_) sanitization with BATT2 (n. of samples: 20 for each mean value).

Room	**Mean value T**_**1 **_**± D.S. (CFU/25 cm**^**2**^**)**	**Mean value T**_**2 **_**± D.S. (CFU/25 cm**^**2**^**)**	Variation (%)	Student's t	p
**A**	0.485 ± 0.972	0.364 ± 1.00	-25	0,273	0,7874

**B**	3.076 ± 7.799	0.28 ± 0.04	-91.1	1.605	0,1167

**C**	2.6 ± 7.87	0.56 ± 0.094	-78.5	0.818	0,4183

**Table 3:** Differences among mean staphylococcal counts registered for fan coil units surfaces before (T_1_) and after (T_2_) sanitization with BATT2 (n. of samples: 20 for each mean value).

Room	**Mean value T**_**1 **_**± D.S. (CFU/25 cm**^**2**^**)**	**Mean value T**_**2 **_**± D.S. (CFU/25 cm**^**2**^**)**	Variation (%)	Student's t	p
**A**	0.2 ± 0.7	0.26 ± 1.8	+30	0,93	0,3576

**B**	2.5 ± 6.06	0.07 ± 0.2	-96.8	1,77	0,0835

**C**	2.1 ± 5.8	0.03 ± 0.1	-98.5	1,61	0,1142

Yeasts, moulds, *P. aeruginosa *and *S. aureus *were not isolated in the first or second monitoring phases.

With regard to the air conditioner used as control (room D), microbial counts were similar to those in the other systems during the first monitoring phase, without marked variations (data not shown).

## Discussion

In the past few decades, the problem of microbial contamination of indoor air has become a subject of interest for several researchers; both for the possible effects on health and for the control measures to limit these effects [8,19]. Exposure to microbial pollutants is in fact related to many negative consequences, such as infectious diseases, toxic effects, allergies, and asthma [4,7,19].

Besides structural solutions and physical systems, several chemical substances with disinfectant power have been employed to reduce these effects, especially in cooling water systems, which represent an ideal habitat for multiplication of amoebas, *Legionella *spp. and *Aspergillus *spp. [4,7,12,13,20].

In the present study, the biodetergent BATT2, a natural product based on brown seaweed extracts, was utilized for the disinfection of fan coil units from four air conditioning systems, to verify its effectiveness. In a previous study, the product was tested *in vitro*, by evaluating its biocidal effect on several species of multidrug-resistant microorganisms isolated in hospital setting. That research showed that BATT2 was efficacious in reducing both microbial and mycotic counts, even when organic substances were added [15].

Here, when used at a 25% concentration, the biodetergent was able to reduce microbial pollution of air and surfaces at 37°C or 22°C.

For the fan coil units of rooms B and C, the mean percentage reduction in mesophilic microbial count and staphylococcal count on surfaces was close to 100%. Mesophilic microbial counts in the air supplied by the three air conditioners tested decreased after treatment with BATT2, with percentages lower than those registered for surfaces, but always higher than 50%. This minor reduction could have been due to the presence of operators in the rooms during the sampling time [17].

For the conditioning systems in room A, the decrease in mesophilic microbial count for surfaces was lower than that for the other systems, and a small increase in staphylococcal count was observed after sanitization. For this room, it is possible that factors other than those mentioned above, related to the use of this environment, were responsible for these results. In fact, the high number of persons who normally attend the reception room was probably related to the high levels of air contamination that were found in this room. As for the surfaces, however, it must be underlined that the observed variations regarded low counts and differences were not statistically significant. Moreover, it has to be noted that the assessment of cleaning conditions of fan coil units was not the aim of this study, then the number of samples analyzed was not wide enough to make any statement about this subject. Anyway, results from air conditioners with higher baseline contamination could be helpful to assess the true value of the systems under investigation.

Finally, the biodetergent BATT2 was able to considerably reduce the psychrophilic count. The presence of psychrophilic microorganisms in indoor environments is often related to high levels of dampness and dust, low air quality and poor ventilation, which favor the growth of bacteria and moulds [7].

Several studies have reported that the prevalence of SBS is higher in air-conditioned than in naturally ventilated buildings, and this supports the employ of systems for natural ventilation in hospital settings [4,7,21-23]. This association is due to the emission of chemical and microbiological pollutants by air conditioners and ventilation systems. Microbial growth on damp surfaces, such as that in air conditioners, represents one of the main bio-contamination sources in old buildings [7]. Therefore, poor and inadequate maintenance of this equipment is an important risk factor for the health of those who stay in conditioned/ventilated environments [24]. Thus, correct and systematic sanitization of all the components of conditioning systems appears to be necessary, mainly in hospitals, which accommodate persons who are particularly susceptible to environment-related risks.

In this study, the biocidal detergent CLEANER COND. B was tested for the cleaning and disinfection of fan coil units from air conditioners located in hospital environments with a mean degree of risk, which are routinely treated with alcool-based detergents. Although results were not significant, it was able to reduce the level of bio-contamination on the analyzed surfaces. Moreover, a significant improvement in the microbiological quality of air supplied by these systems was observed.

On the basis of the present and previous experience [15], BATT2 can be considered suitable for the sanitization of fan coil units. In the future, its use will be compared with that of other type of detergents/disinfectants commonly employed in hospital settings. A greater sample size could allow to verify the results obtained in the present experience and their significance.

It should be noted that prevention of the risks related to the use of conditioning systems cannot be based only on the effectiveness of the detergent employed, although it is fundamental. The correct management of these systems must include periodical maintenance interventions with sanitization procedures based on rigorous protocols and verifiable through proper monitoring programs [4,5,25,26].

## Competing interests

The authors declare that they have no competing interests.

## Authors' contributions

GL conceived of the study and carried out its design. MB performed the assays and drafted the manuscript. FG and VDO drafted and edited the manuscript. MN analyzed the results of tests. MT participated in the design and supervision of the study. All authors have read and approved the final manuscript.
